# Prevalence of Fatigue and Associations With Depression and Cognitive Impairment in Patients With CADASIL

**DOI:** 10.1212/WNL.0000000000213335

**Published:** 2025-01-16

**Authors:** Amy A. Jolly, Success Anyanwu, Fatemeh Koohi, Robin G. Morris, Hugh S. Markus

**Affiliations:** 1 Department of Clinical Neurosciences, University of Cambridge, United Kingdom;; 2 School of Clinical Medicine, University of Cambridge, United Kingdom; and; 3 King's College Institute of Psychiatry, Psychology and Neuroscience, London, United Kingdom.

## Abstract

**Background and Objectives:**

Fatigue is a common and disabling symptom in cerebrovascular disease and has been associated with white matter damage, but the underlying disease mechanisms are poorly understood. Cerebral autosomal dominant arteriopathy with subcortical infarcts and leukoencephalopathy (CADASIL) is the most common genetic form of stroke and causes a cerebral small vessel disease arteriopathy with white matter ischemia. We determined the prevalence of fatigue in CADASIL, the factors associated with it, and its relationship with both depression and cognitive impairment.

**Methods:**

Prospectively recruited genetically confirmed patients with CADASIL were assessed using the Fatigue Severity Scale. The prevalence of fatigue in CADASIL was compared with that of healthy controls from the community. We determined associations between fatigue and clinical features, cardiovascular risk factors, MRI parameters, cognition, and depression. Cognition was measured using the Brief Memory and Executive Test (BMET) and depression using the Geriatric Depression Scale (GDS). Mediation and path analyses were performed to determine relationships between fatigue, depression, and cognitive impairment.

**Results:**

One hundred seventy-four patients with CADASIL (mean age [SD] of 51.3 [12.30] years, 59.66% female) and 50 healthy controls were included in the analysis (mean age [SD] of 51.42 [12.58] years, 38.0% female). Fatigue was present in 51.7% of patients with CADASIL and was almost 5 times more common than in controls (OR: 4.99, 95% CI [2.28–10.95], *p* < 0.001). There was no association of fatigue with history of stroke or MRI parameters including white matter hyperintensity lesion volume. Logistic regression showed both GDS total score (OR: 1.11 [1.05–1.17], *p* = 0.0002) and BMET total score (OR: 0.86 [0.75–0.98], *p* = 0.02) to be predictors of fatigue. Fatigue, depression, and cognition were frequently comorbid. Mediation analysis showed depression to have a greater effect on fatigue prevalence than cognitive impairment. Path analysis confirmed depression to be the largest predictor of fatigue and found this relationship to be bidirectional.

**Discussion:**

Fatigue was present in over half of the patients with CADASIL. Depression and cognition were the main predictors of fatigue, and all 3 symptoms were frequently comorbid. The relationship between depression and fatigue was the strongest and was bidirectional. This suggests targeting depressive symptoms may have benefit in fatigue management.

## Introduction

Fatigue is a common symptom after stroke,^1,2^ estimated to affect up to 50% of stroke survivors.^3,4^ Despite this, the underlying mechanisms are poorly understood, leading to few effective therapies.^5^ Fatigue is also commonly seen in neurologic diseases affecting the white matter, such as multiple sclerosis, and has been reported to be common in cerebral small vessel disease (SVD).^6,7^ A recent systematic review of neuropsychiatric features in SVD found white matter hyperintensities (WMHs), a characteristic MRI feature of SVD, to be associated with fatigue.^7^

Cerebral autosomal dominant arteriopathy with subcortical infarcts and leukoencephalopathy (CADASIL) is the most common monogenic form of stroke.^8,9^ CADASIL causes a small vessel arteriopathy resulting in SVD with prominent involvement of the white matter and the presence of both lacunar infarcts and confluent WMHs.^9^ Clinical features of CADASIL include migraine with aura, stroke, and cognitive impairment and dementia.^8,9^ Case studies have reported fatigue as a symptom in CADASIL,^10-12^ but little systematic research has investigated its prevalence and risk factors. Investigation of fatigue in CADASIL may also give insights into the mechanism of the association between fatigue and white matter disease.

Fatigue is highly associated with other neuropsychiatric symptoms of which the most commonly cited is depression, with the 2 symptoms being reported to be frequently comorbid.^2,13^ Cognitive impairment has also been associated with the presence of fatigue after stroke.^2^ Notably, depression and cognitive impairment are also common symptoms in CADASIL.^8,14,15^ How fatigue, depression, and cognitive impairment inter-relate and whether either depression or cognitive impairment plays a role in the pathogenesis of fatigue remain uncertain.

To investigate this further, we determined the prevalence of fatigue in CADASIL and its association with clinical and demographic factors, as well as MRI markers of SVD. To better understand the association between fatigue and neuropsychiatric symptoms, we also determined the relationship of fatigue with depression and cognitive impairment and examined the inter-relationships between the 3 using path analysis.

## Methods

### Participants

#### CADASIL Population

The CADASIL population analyzed were patients recruited to the UK Familial Cerebral Small Vessel Disease (FSVD) study, a prospective cohort study recruiting those with suspected monogenic SVD from 5 UK centers (mentioned in Acknowledgments). Medical history and family history were collected, as well as the original clinical MRI scans.

#### Control Population

Patients with CADASIL were compared with controls recruited as part of the Fatigue in Healthy Controls Study. Controls were recruited from the community by way of advertisement flyers through social media, local groups, and charities. Mood testing and cognitive testing were conducted using the same measures performed in the CADASIL cohort.

### Measures

#### Demographics and Medical History

Demographics including age, sex, and vascular risk factors were collected for both CADASIL cases and controls. For CADASIL participants, medical history was also collected including commonly reported symptoms in CADASIL: migraine, stroke, encephalopathy, psychiatric disorders, and cognitive decline. If any features were present, age at occurrence and details of the symptom were recorded.

#### Fatigue Severity Scale (FSS)

The FSS is a brief measure of fatigue that assesses the influence of fatigue on daily behaviors.^16^ The measure has 9 questions in which respondents are required to indicate on a 7-point Likert scale ranging from ‘strongly disagree’ to ‘strongly agree’ in response to statements such as ‘I am easily fatigued.’^16^ The FSS also includes the Visual Analog Fatigue (VAF) Scale, which asks patients to place their overall fatigue on a scale of 0–10. To classify fatigue, the scores of the first 9 questions are averaged, and a score of ≥4 has been taken to indicate significant fatigue.^17,18^

#### Geriatric Depression Scale (GDS)

The GDS is a short depression-screening questionnaire that requires patients to answer yes/no to 30 statements.^19^ A score of 0–9 indicates within normal range, 10–19 mild depressive symptoms, and 20–30 severe depressive symptoms.^19^ The GDS has successfully been used in previous CADASIL population studies.^20^ The GDS total score can subdivided into apathy, anxiety, and fatigue subscales.^21^ The maximum score on the anxiety and apathy subscales is 10 while the maximum score on the fatigue subscale is 9.^21^ Higher scores indicate higher reported levels of the feature measured by the subscale.^21^

#### Brief Memory and Executive Test (BMET)

The BMET is a brief cognitive screening tool designed for, and shown to be sensitive to, cognitive impairment in SVD.^22,23^ The total maximum score on the BMET is 16, which can be broken down into 2 subscales: Executive Functioning and Processing Speed (EF/PS) (maximum score of 8) and Orientation and Memory (O/M) (maximum score of 8) as previously described.^22,23^ A previously validated cutoff score of ≤13 was taken to indicate vascular cognitive impairment (VCI).^23^

### Neuroimaging Features

Original clinical MRI scans of patients with CADASIL were analyzed. If a patient had multiple scans, the most recent at the time of recruitment was used. T1-weighted, gradient echo (GE), susceptibility-weighted imaging (SWI), diffusion-weighted imaging (DWI), and fluid-attenuated inversion recovery (FLAIR) sequences were required for analysis of neuroimaging features.

#### Normalized Brain Volume

SIENAX (from FSL software, fsl.fmrib.ox.ac.uk^24,25^) was used to estimate total brain volume, subdivided into white matter and gray matter, with T1 sequences. Brain volumes accounted for estimated skull size with a visual scaling factor (VSF).

#### Cerebral Microbleeds

Cerebral microbleeds (CMBs) were measured using the Brain Observer Microbleed Rating Scale^26^ on GE or SWI sequences. CMBs were rated by an expert neurologist rater.

#### Lacune Count

A neurologist expert rater counted the number of lacunar infarcts including both acute infarcts using DWI and old cavitated lacunar infarcts using T1 and FLAIR sequences. A lacune was defined as a subcortical infarct between 3 and 15 mm in diameter.^27^

#### Lesion Volume

Trained raters quantified WMHs using the semiautomated contouring tool in Jim image analysis software, version 8 (Xinapse Systems^28^). WMHs were defined as areas on FLAIR images showing increased signal. WMHs were normalized for skull size using the VSF produced by SIENAX. Inter-rater agreement between the 2 trained raters was good with an intraclass correlation coefficient of 0.96, calculated from a subset (n = 10) of the data.

### Standard Protocol Approval and Consent

The FSVD Study was approved by the East of England Cambridge Central Research Ethics Committee (16/EE/0118). The Fatigue in Healthy Controls Study was approved by the University of Cambridge Psychology Research Committee (PREC.2022.054). All participants gave written informed consent. For those without capacity in the FSVD Study, a consultee gave written informed consent.

### Statistical Analyses

Participants were classified as having fatigue if their average FSS score was ≥4, as previously defined.^17,18^ Depressive symptoms were assessed as a continuous variable using the GDS total score and also binarized into depressed and not depressed by collapsing the mild and severe depressive groups into the depressive group (score of 10+). The GDS total score was also subdivided into anxiety, apathy, and fatigue subscales as previously described.^21^ Cognition was assessed as a continuous variable using the BMET total score and the EF/PS and O/M subscales. VCI was determined by the BMET using a cutoff of ≤13 as previously described.^22,23^

Differences in demographics and clinical characteristics, cognitive test scores, and fatigue and GDS scores were compared between the subgroups (patients with CADASIL and controls; patients with CADASIL with and without fatigue), using the χ^2^ test, *t* tests, and logistic regression, where appropriate. All regression analyses were controlled for age and sex. Any significant associations were entered into logistic regression to identify independent predictors of fatigue prevalence.

Owing to non-normal distributions, all MRI parameters were normalized. Brain volume was normalized using the square transformation. Lesion volume and lacune count were normalized using the square root transformation, and CMBs were normalized using a logarithm transformation. Only 117 participants had sequences for analysis of CMBs; therefore, these were excluded from the main analysis and analyzed separately to retain sample size. MRI parameters were compared between fatigued and nonfatigued patients with CADASIL using logistic regression, while controlling for age and sex.

Correlations between the average FSS score and cognitive behavioral symptoms (cognitive score and depression score) were performed using the Spearman rank test. Venn diagram was used to display the comorbidity of cognitive, depressive, and fatigue symptoms.

Mediation analyses were then performed for each symptom using the binarized measures.

Finally, path analysis was performed using the R package “Lavaan” (version 0.6-15)^29^ to identify a model able to best explain direct and indirect effects of cognition, depression, and fatigue on one another. Multiple models were modeled, and only the best fitting was reported and displayed graphically. A sample of 153 participants with complete data were selected for this.

Model fit was assessed using the model test statistic and 3 measures of model fit.^30^ For the model to be accepted as accurate, the *p* value for the χ^2^ test must be *p* ≥ 0.05.^30^ The root mean square error of approximation (RMSEA) and 90% CIs were also checked; the RMSEA must be 0.05–0.08 (acceptable) or ≤0.05 (excellent).^31^ The comparative fit index (CFI) is a score from 0 to 1, with a cutoff of ≥0.95 used to determine good model fit.^30,32^ Finally, the standardized root mean residual (SRMR) value was assessed, with a value of ≤0.08 used to determine good model fit.^32^ Robust versions of the model fit statistics were used where possible to account for non-normal data.

## Results

A total of 359 patients have been recruited to the FSVD Study to date; of these, 251 had genetically confirmed CADASIL with typical cysteine-changing variants and were included. Assessment of fatigue was only added as an amendment part way through the study; as such, 174 had fatigue testing and were included in analyses. Of these 174, 170 had depression testing and 169 had cognitive testing (eTable 1). Fifty healthy controls with no history of serious medical history were included.

### Comparison of Patients With CADASIL and Controls

There were no significant differences in demographics or vascular risk factors between the CADASIL and the control groups (Table 1). The CADASIL group had lower scores on the BMET EF/PS index, but not on the O/M index^23^ (Table 1). The CADASIL group scored higher on the FSS and GDS compared with the control group (Table 1).

**Table 1:** Comparison of Demographics and Clinical Features of Patients With CADASIL and Controls

	Population		
	CADASIL (n = 174)	Controls (n = 50)	Comparison
Demographic and cardiovascular risk factors			
Age at clinic (mean, SD, min-max)	51.43, 12.47, 22–77	51.42, 12.58, 19–72	*p* = 1.00
Sex (% male)	71 (40.34)	19 (38.0)	*p* = 0.89
History of hypertension (% yes)	45 (25.57)	9 (18.0)	*p =* 0.27
Diabetes mellitus (% yes)	11 (6.25)	7 (14.0)	*p =* 0.08
History of ever smoking (% yes)	59 (33.52)	15 (30.0)	*p =* 0.63
Modified Rankin Scale score (median, IQR, range)^c^	0, 1, 0–4	0, 0, 0–0	*p =* 0.98
Cognitive measures			
BMET total score (median, IQR, min-max)	14, 4, 2–16	15, 3, 8–16	*p* = 0.02^a^
EF/PS score (median, IQR, min-max)	8, 2, 0–8	8, 0, 3–8	*p* = 0.007^b^
O/M score (median, IQR, min-max)	7, 3, 0–8	7, 2, 2–8	*p* = 0.21
VCI on BMET (% yes)^c^	69 (40.35)	13 (28.26)	*p* = 0.14
Depression measures			
History of depression (% yes)	49 (27.84)	7 (14.0)	*p =* 0.05^a^
GDS total score (mean, SD, min-max)^c^	9.97, 7.53, 0–30	4.60, 3.97, 0–18	*p* < 0.001^b^
Fatigue measures			
FSS fatigue	84 (48.28)	9 (18.0)	*p* < 0.001^b^
Average FSS score (mean, SD, min-max)	4.09, 1.69, 1–7	2.86, 1.06, 1.11–5.11	*p* < 0.001^b^

Abbreviations: BMET = Brief Memory and Executive Test; EF/PS = Executive Functioning and Processing Speed; FSS = Fatigue Severity Scale; GDS = Geriatric Depression Scale; O/M = Orientation and Memory; VAF = Visual Analog Fatigue; VCI = vascular cognitive impairment.

Excluding age and sex, all *p* values are adjusted for age and sex. Only those with cysteine-changing variants were included.

a *p* < 0.05.

b *p* < 0.01.

c Indicates missing data, in eTable 1.

### Prevalence of Fatigue

In the CADASIL group, 51.7% (90/174) were classed as having fatigue compared with only 8.0% (4/50) of the controls. On logistic regression, patients with CADASIL were about 5 times more likely to have fatigue on the FSS than controls (OR: 4.99, 95% CI [2.28–10.95], *p* < 0.001).

### Between-Group Comparison

Fatigue was present in the CADASIL group in all age groups (eFigure 1), and there was no difference in age between the fatigued and nonfatigued CADASIL groups (Table 2). Additional Spearman correlation showed no correlation between age and the average fatigue score on FSS (*ρ* = 0.12, *p* = 0.11).

**Table 2:** Comparison of Demographics, Clinical History, Cognitive Behavioral Symptoms, and MRI Parameters Between Those With and Without Fatigue on the FSS

	FSS fatigue		
	No (n = 84)	Yes (n = 90)	Comparison
Demographics and vascular risk factors			
Age at BMET (mean, SD, min-max)	49.73, 12.42, 22–71	52.88, 12.49, 22–77	*p* = 0.09
Sex (% male)	38 (45.24)	32 (64.44)	*p* = 0.25
EGFr grouping (median, IQR, min-max)	4, 8.25, 1–31	4, 3, 2–31	*p* = 0.15
EGFr grouping 1–6 (%)	56 (66.67)	68 (75.56)	*p* = 0.11
History of hypertension (% yes)	19 (22.62)	25 (27.78)	*p =* 0.57
Diabetes mellitus (% yes)	3 (3.57)	8 (8.89)	*p =* 0.16
History of ever smoking (% yes)	28 (33.33)	29 (32.22)	*p =* 0.79
Medical/clinical history			
History of migraine (% yes)	61 (72.62)	67 (74.44)	*p =* 0.90
History of migraine with aura (% yes)	58 (69.05)	62 (68.89)	*p =* 0.81
History of TIA (% yes)	8 (9.52)	6 (6.67)	*p =* 0.20
History of stroke (% yes)	30 (35.7)	36 (40.0)	*p =* 0.84
Recurrent stroke (% yes)	4 (4.76)	10 (11.11)	*p =* 0.24
History of encephalopathy (% yes)	4 (4.76)	8 (8.89)	*p =* 0.40
History of epilepsy (% yes)	10 (11.90)	8 (8.89)	*p =* 0.54
Behavioral symptoms^c^			
History of psychiatric disorder (any (% yes))	21 (25.0)	43 (48.31)	*p =* 0.003^b^
History of depression (% yes)	16 (19.05)	32 (35.56)	*p =* 0.02^a^
GDS total score (median, IQR, min-max)	5, 6.5, 0–30	12, 8, 0–29	*p* < 0.001^b^
GDS apathy (median, IQR, min-max)	1, 3, 0–10	3, 5, 0–10	*p* < 0.001^b^
GDS anxiety (median, IQR, min-max)	1, 3, 0–10	3, 4, 0–10	*p* < 0.001^b^
GDS fatigue (median, IQR, min-max)	2, 3, 0–8	5, 3, 0–9	*p* < 0.001^b^
Cognitive symptoms^c^			
History of cognitive impairment (% yes)	8 (9.52)	29 (32.22)	*p =* 0.001^b^
BMET total score (median, IQR, min-max)	15, 3, 7–16	13, 4, 2–16	*p =* 0.001^b^
EF/PS score (median, IQR, min-max)	8, 1, 0–8	7, 2, 0–8	*p =* 0.03^a^
O/M score (median, IQR, min-max)	7.5, 2, 3–8	6, 3, 0–8	*p =* 0.001^b^
VCI on BMET (% yes)	22 (26.83)	45 (51.72)	*p =* 0.001^b^
Neuroimaging (mean, SD, min-max)^c^			
Total brain volume (mm^3^)	1,605,907.48, 146,626.35, 1,116,760.97–1,905,869.62	1,587,384.69, 157,759.07, 1,050,256.91–1962324.50	*p =* 0.81
White matter volume (mm^3^)	839,916.35, 119,901.36, 287,863.70–1130497.80	811,109.66, 155,801.54, 208,052.97–1,031,950.31	*p =* 0.32
Gray matter volume (mm^3^)	765,740.41, 91,976.03, 498,596.65–998,105.20	776,276.27, 140,284.97, 500,548.92–1,319,400.45	*p =* 0.34
WMH volume (mm^3^)	80,837.17, 66,659.24, 0–356561.15	88,366.52, 65,193.32, 0–277447.07	*p =* 0.70
CMB count	3.97, 12.04, 0–80	10.47, 35.55, 0–197	*p =* 0.48
Lacune count	2.41, 3.57, 0–18	3.15, 4.20, 0–19	*p =* 0.47

Abbreviations: BMET = Brief Memory and Executive Test; CMB = cerebral microbleed; EF/PS = Executive Functioning and Processing Speed; EGFr = epidermal growth factor-like repeat; GDS = Geriatric Depression Scale; O/M = Orientation and Memory; VCI = vascular cognitive impairment; WMH = white matter hyperintensity.

Excluding age and sex, all *p* values are adjusted for age and sex. All brain volumes and WMH volume were normalized to account for skull size.

a *p* < 0.05.

b *p* < 0.01.

c Indicates missing data, in eTable 1.

Within the CADASIL group, the presence of fatigue was associated with a higher prevalence of depression and lower cognitive scores, including for both EF/PS and O/M subdomains (Table 2). There were no other differences in demographics or clinical features including stroke prevalence between the fatigued and nonfatigued groups (Table 2). Within the CADASIL cohort, there were no differences in any MRI parameter (brain volume, WMH volume, lacune count, or CMB count) between those with and without fatigue (Table 2).

### Logistic Regression

Significant variables (BMET score and GDS score) were entered into logistic regression while controlling for age and sex.

On logistic regression, while controlling for age and sex, both GDS total score (OR: 1.11 [1.05–1.17], *p* = 0.0002) and BMET total score (OR: 0.86 [0.75–0.98], *p =* 0.02) were predictors of FSS fatigue grouping.

### Relationships Among Fatigue, Depression, and Cognitive Behavioral Symptoms

**Figure 1:**
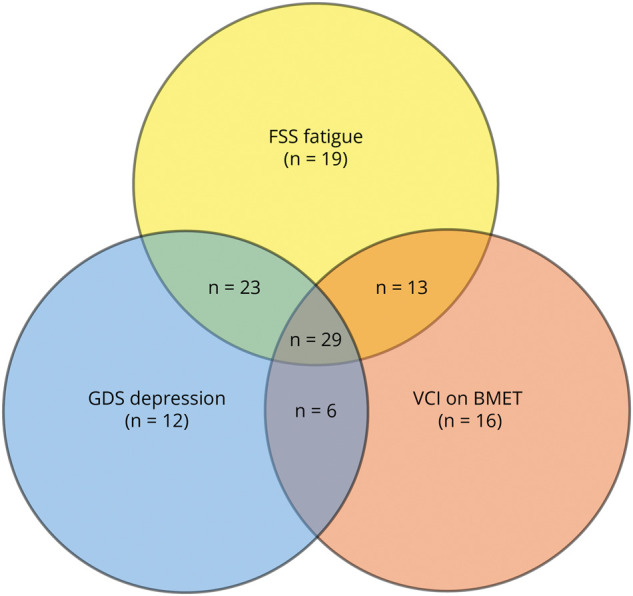
Venn Diagram Showing Comorbidities Between Cognitive Impairment on BMET, Depression on the GDS, and Fatigue on the FSS in the CADASIL Cohort BMET = Brief Memory and Executive Test; FSS = Fatigue Severity Scale; GDS = Geriatric Depression Scale.

There was much comorbidity between binarized GDS depression, BMET VCI, and FSS fatigue symptoms (Figure 1). The most common presentation was with all 3 symptoms (n = 29, 16.67%), followed by comorbid fatigue and depression (n = 23, 13.22%) (Figure 1). There was a significant correlation between the average fatigue score and the GDS total score (Spearman *ρ* = 0.45, *p* < 0.001) and BMET total score (*ρ* = −0.31, *p* < 0.001). There was also a significant correlation between the GDS total score and BMET total score (*ρ* = −0.28, *p* < 0.001).

### Mediation Analyses

**Figure 2:**
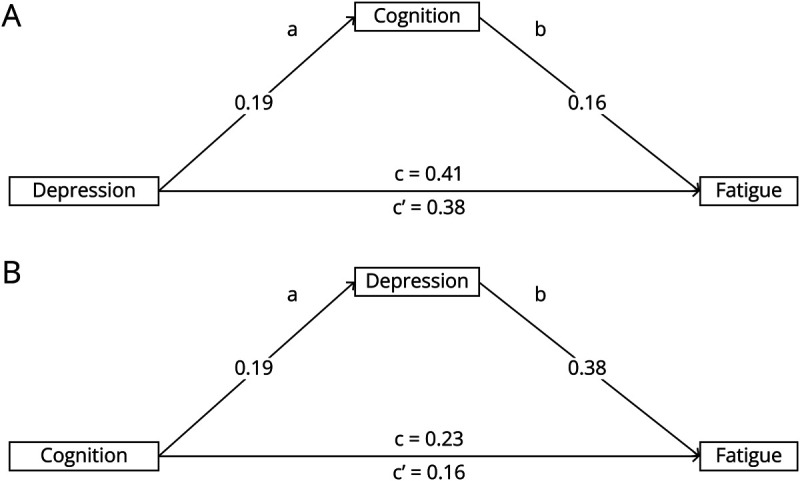
Direct and Total Effects of GDS Depression (A) and BMET VCI (Cognition) (B) on FFS Fatigue in the Mediation Models c represents the effect of one variable on another (total effect), whereas c’ represents this effect while further removing the effects of the third mediated variable (direct effect). BMET = Brief Memory and Executive Test; FSS = Fatigue Severity Scale; VCI = vascular cognitive impairment.

Mediation analysis was performed to explore the effects of depression and cognition on fatigue and to investigate whether the effect of depression on fatigue was mediated by cognition and, conversely, whether the effect of cognition on fatigue was mediated by depression. The analysis showed depression to have a larger direct effect on fatigue presence than cognitive impairment (Figure 2; c’ = 0.38 vs cognitive impairment: c’ = 0.16). The effect of depression on fatigue was direct and not mediated through cognition (ab = 0.03, 95% CI [0.00–0.08]) (Figure 2A). By contrast, the effect of VCI on fatigue prevalence was mediated through depression (ab = 0.07, 95% CI [0.01–0.15]) (Figure 2B).

### Path Analyses

#### Model Fit

**Figure 3:**
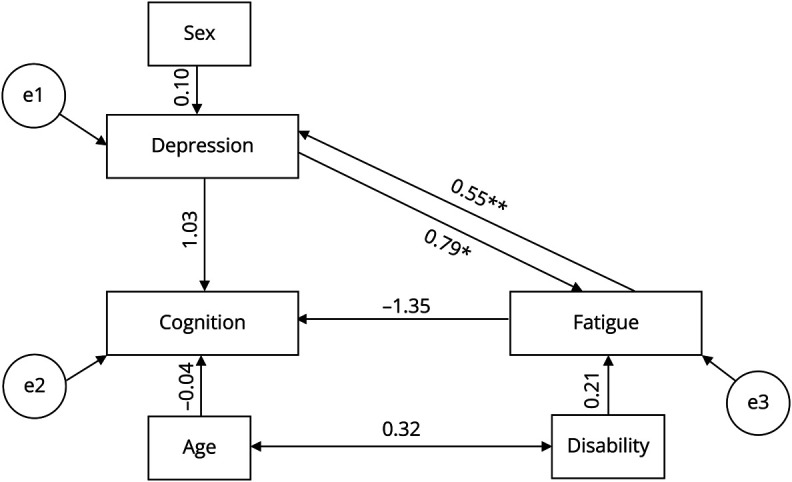
Path Diagram of Relationships Among Cognition, Depression, and Fatigue *p < 0.05, **p < 0.01. Rectangles denote variables included in the path model. Single-headed arrows represent hypothesized causal pathways, with adjacent numbers indicating standardized regression coefficients. Double-headed arrows represent covariance between variables. e1, e2, and e3 denote the error terms or residuals for each endogenous variable (GDS score, average FSS score, and BMET total score), representing the variance unexplained by the model's predictors. BMET = Brief Memory and Executive Test; FSS = Fatigue Severity Scale; GDS = Geriatric Depression Scale.

The derived model is shown in Figure 3. All necessary model fit values were met; the χ^2^ *p* value was 0.29, exceeding 0.05 in line with recommendations.^30^ CFI and robust CFI were both 0.99, indicating good model fit^30,32^; the robust RMSEA was 0.04 [0.00–0.13] indicative of excellent model fit,^31^ and the SRMR was 0.03, again below the threshold of ≤0.08 and indicative of good model fit.^32^

#### Direct and Indirect Effects

In the model, there was a significant positive direct effect of the average FSS score on the GDS score (*β* = 0.55, *p* = 0.004) (Figure 3, Table 3). This relationship was bidirectional, and there was also a significant and positive direct effect of the GDS score on the average FSS score (*β* = 0.79, *p* = 0.05) (Figure 3, Table 3). The effect of depression on fatigue was slightly stronger than vice versa (Figure 3, Table 3). There was a significant total effect of fatigue on cognition, but this was not explained by the direct or indirect effects of fatigue on cognition (Table 3).

**Table 3:** Direct, Indirect, and Total Effects Estimated Using the Path Model

Endogenous variables	Predictors	Estimate of direct effect		*p* Value	Estimate of indirect effect		*p* Value	Estimate of total effect		*p* Valu*e*
		Unstandardized	Standardized		Unstandardized	Standardized		Unstandardized	Standardized	
GDS score	Average FSS score	2.326	0.549	0.004^a^	NA	NA	NA	2.326	0.549	0.004^a^
	BMET total score	NA	NA	NA	NA	NA	NA	NA	NA	NA
	Sex	1.513	0.103	0.186	NA	NA	NA	NA	NA	NA
Average FSS score	GDS score	0.186	0.790	0.054^a^	NA	NA	NA	0.186	0.790	0.054^a^
	BMET total score	NA	NA	NA	NA	NA	NA	NA	NA	NA
	MRS score	0.415	0.214	0.068	NA	NA	NA	NA	NA	NA
BMET total score	Average FSS score	−2.335	−1.348	0.152	0.978	0.594	0.489	−1.358	−0.783	0.003^a^
	GDS score	−0.010	−0.040	0.588	−0.435	−1.064	0.361	−0.015	−0.036	0.930
	Age	0.420	1.028	0.436	NA	NA	NA	NA	NA	NA

Abbreviations: BMET = Brief Memory and Executive Test; FSS = Fatigue Severity Scale; GDS = Geriatric Depression Scale.

Note: n = 153. NA denotes the path not modeled. Where the direct path was the only path modeled, this is also the total effect. Standardized coefficients (mean = 0, SD = 1) allow for comparison across variables while unstandardized coefficients retain the original measurement scale of each variable.

a Significant path.

#### Correlations

There was a significant positive correlation between the modified Rankin Scale score (disability) and age (*p* < 0.001) (Figure 3, eTable 2). A significant positive correlation was also observed between the fatigue score and depression score (*p* = 0.04) (eTable 2).

### Cognitive Subdomains

Additional analyses were undertaken to assess the relationship between the cognitive subdomains and fatigue.

On mediation testing the effects of EF/PS subscale score on fatigue with depression as a mediator, the EF/PS score had a total effect on fatigue (c = −0.2, *p* = 0.02), which was lost when removing the effects of depression (c’ = −0.01, *p* = 0.21). There was also an indirect effect of the EF/PS subscale score on fatigue through depression (ab = −0.01, 95% CIs [-0.2, −0.02]).

A further mediation assessing effects of O/M subscale score and depression on fatigue showed an effect of the O/M subscale score on fatigue (eFigure 2A: total effect (c = −0.27, *p* < 0.001) and a direct effect (c' = −0.22, *p* = 0.003)), which was not mediated through depression.

A separate mediation showed no significant total (c = −0.13, *p* = 0.12) or direct (c = −0.01, *p* = 0.92) effect of the O/M score on depression. On the contrary, a stronger effect of the EF/PS subscale score on depression was observed (c = −0.24, *p* = 0.003), which persisted after removing effects of fatigue (c’ = −0.16, *p* = 0.04) and was also mediated through fatigue (ab = −0.08, 95% CIs [-0.15, −0.01]) (eFigure 2B).

## Discussion

In this large cohort of patients with CADASIL, we found that fatigue was common, being present in just over half of the participants. This was 5 times more common than in controls. The major predictors of fatigue were depressive symptoms and cognitive impairment, with depression being the strongest predictor of fatigue. Path analysis indicated that the relationship between depression and fatigue was bidirectional.

The prevalence of fatigue in this cohort is similar to estimates for poststroke fatigue of around 50%.^3,4^ Despite its high prevalence, there is little to no research establishing the prevalence, and predictors, of fatigue in CADASIL cohorts; our findings emphasize the importance of this symptom in this patient group. Studies have repeatedly shown the major impact of fatigue on patient quality of life in stroke.^33,34^

It has been suggested that disease severity, and particularly the presence of stroke, may be associated with fatigue. However, while CADASIL itself was associated with increased fatigue, we found no association between fatigue and brain markers of disease severity within the CADASIL cohort. In particular, there was no association with a history of stroke or with lacune count on MRI. We also found no association with WMH volume. Some previous studies have reported an association between WMH severity and fatigue in SVD^7^; however, a recent systemic review found no robust evidence supporting this association and found few correlations between neuroimaging features and the presence of poststroke fatigue.^35^

We found a robust association between depression and fatigue within the CADASIL cohort. This was seen both when binarized scores were used (logistic regression and mediation analyses) and when continuous scores were used (path analysis). Depression has been frequently reported in those with fatigue, and the 2 symptoms are known to be comorbid.^1,13^ Likewise, research has found symptoms within depression and fatigue to be highly correlated, with overlap in domains such as insomnia and poorer concentration.^36^ Our results suggest that the relationship between fatigue and depression is bidirectional, further supporting research highlighting that comorbid depression and fatigue may exacerbate one another and worsen outcomes.^37^ Such findings raise the possibility that the fatigue associated with SVD is not caused directly by the disease process, but in an indirect effect of a syndrome including coexisting fatigue and depression. It follows that, in theory, treatment of depression should alleviate symptoms of fatigue by reducing the effects of the bidirectional relationship. However, a recent review concluded that antidepressants had limited impact on reducing fatigue.^38^ Recent research has suggested that the mechanism of fatigue may be heterogeneous with a number of different mechanisms underlying fatigue^1,5^; therefore, it is possible that depression may play a role in only some cases; more accurate characterization of the different fatigue subtypes and selection of those in whom depression plays a role may allow better treatment responses. In addition, the use of fatigue management may play a role in alleviating the symptoms associated with SVD.

Our results also showed that fatigue was increased in those patients with CADASIL with cognitive impairment. Assessment of cognitive subdomains showed that the EF/PS score was more closely linked to depression than to fatigue. It has been hypothesized that reduced executive functioning and processing speed may be part of presentation in depression.^39^ Similarly, both EF/PS and depression have been associated with the extent of white matter damage in SVD.^40,41^ As such, it follows that these 2 variables would be most closely associated in these analyses. Initial analyses of the group characteristics showed that more patients showed specific impairments in EF/PS; however, O/M mediation analyses indicated that this variable was more associated with fatigue. It can be speculated that, in this instance, increased fatigue reduced attentional resources required for memory encoding and retrieval processes, with memory impairment also common in depression.^42^ Further multivariate analyses differentiating fatigue interactions with different facets of cognition are needed to explore this further in this and other conditions.

Our study has several strengths. It included a large cohort of patients with CADASIL who were prospectively recruited. We collected measures on fatigue, depression, and cognition allowing the inter-relationships between the 3 to be determined. However, it also has limitations. We used clinically acquired MRI scans; therefore, they were not uniform in make of scanner or type of sequences used. We only used one measure of fatigue, the FSS. This has been well validated and widely used in neurologic populations and has been shown to have a reliable single-factor measurement.^43^ However, it is now recognized that fatigue is highly multidimensional and has been associated with a multitude of factors.^1^ Likewise, recent research has suggested that fatigue measures may measure separate aspects of fatigue.^43^ Further investigation with a multidimensional fatigue scale, or indeed, battery of fatigue questionnaires, may better encapsulate the different facets of fatigue.

In summary, we have shown a high prevalence of fatigue in patients with CADASIL. Depression was the major risk factor of fatigue, and path analysis showed a bidirectional relationship between fatigue and depression, suggesting that at least in some cases of fatigue, targeting depression may improve outcome. By contrast, we found no association of clinical features and the extent of white matter damage on MRI with fatigue.

## Glossary

BMET: Brief Memory and Executive Test
CADASIL: cerebral autosomal dominant arteriopathy with subcortical infarcts and leukoencephalopathy
CMBs: cerebral microbleeds
EF/PS: Executive Functioning and Processing Speed
FSS: Fatigue Severity Scale
GDS: Geriatric Depression Scale
NBV: normalized brain volume
O/M: Orientation and Memory
RMSEA: root mean square error of approximation
SRMR: standardized root mean residual
SVD: small vessel disease
VAF: Visual Analog Fatigue
VCI: vascular cognitive impairment
WMHs: white matter hyperintensities
